# HISTÓRIA, CIÊNCIAS, SAÚDE – MANGUINHOS: CELEBRATING 30 YEARS IN 2024

**DOI:** 10.1590/S0104-59702025000100026en

**Published:** 2025-06-16

**Authors:** Marcos Cueto

**Affiliations:** i Science editor, researcher, Casa de Oswaldo Cruz/Fiocruz. Rio de Janeiro – RJ – Brazil

As in previous years, here we present a summary of the journal’s main activities in 2024, corresponding to volume 31 (Cerqueira, Cueto, 2024; Cueto, 2023). This period was marked by the journal’s thirtieth anniversary, the ongoing process of internationalization, and efforts to restructure the editorial staff.

In August 2024, we celebrated 30 years of publication, with a special meeting entitled “Open science and scientific publications in the humanities,” held on August 7 and 8 at the Fiocruz campus in Rio de Janeiro. The event focused on open science, journal assessment, and scientific dissemination, and brought together editors of journals based in Brazil, Mexico and Chile. The sessions and debates were recorded and can be viewed at https://www.youtube.com/watch?v=qYB8V5fbd4U. One problem identified during this meeting (and a challenge for the area in general) is the adoption of open science within the humanities; many researchers in the humanities face difficulties adopting the directives of open science in their work.

For the second year, we continued to publish on continuous publication format. In this system, texts are gathered in a single volume instead of separated into various issues, as we have done in the past. In 2024, *História, Ciências, Saúde – Manguinhos* published 74 texts (in all sections), 18 of which were bilingual. Brazil still predominates among institutional affiliations of lead authors, but we also received contributions from Portugal, Spain, Argentina, Chile, the United States, Mexico, Uruguay, Peru, Colombia and Russia. Notable articles included texts related to the hundredth anniversary of the Instituto Nacional de Saúde da Mulher, da Criança e do Adolescente Fernandes Figueira, a center for teaching, care, and research that has been linked to Fiocruz since 1970 (Teixeira, Velasquez de Souza, 2024). During the second half of 2024 we published a supplement entitled “The evolution of hospital and health governance: between local and global” (https://www.scielo.br/j/hcsm/i/2024.v31suppl1), which presented in-depth, multifaceted analysis on the evolution of hospital management, edited by acclaimed guest editors Isabel Amaral, Alexandra Esteves and Céline Paillette.

Members of our team participated in a workshop held by the Instituto de Comunicação e Informação Científica e Tecnológica em Saúde at Fiocruz in June 2024 on expanding understanding of how the Open Journal Systems platform works; we will deploy this platform this year. We were also present at meetings of the Scientific Electronic Library Online (SciELO), Associação Brasileira de Editores Científicos [Brazilian Association of Science Editors], and Editors Forums for Fiocruz and the Associação Nacional de História [National Association of History]. One of the most relevant initiatives discussed at these meetings was the SciELO Acessibilidade project (https://www.scielo.br/about/gt-acessibilidade), which proposes changes to make scientific publication more inclusive.

In collaboration with the Communications Department at Casa de Oswaldo Cruz and the History of the Sciences and Health Library, we worked with a fellowship recipient dedicated to analyzing the journal’s metrics and creating a methodology to monitor data from *História, Ciências, Saúde – Manguinhos*’s blog , as well as strategies for disseminating our materials on social media.

Hard efforts by the editorial staff resulted in the training of new editing professionals and updating to the reference materials used in the revision process, at the same time that articles were published. We also implemented a new step in the final preparation of manuscripts, which consists of returning the page proofs to authors for a final check, in order to reinforce the transparency of our editing process.

In July 2024, *História, Ciências, Saúde – Manguinhos* submitted a request for funding from the Conselho Nacional de Desenvolvimento Científico e Tecnológico and the Coordenação de Aperfeiçoamento de Pessoal de Nível Superior, which was approved. Over the course of the year, executive editor Roberta Cerqueira participated in two relevant mini-courses. The first, entitled “Historiography of science: production, methodologies, and sources in the digital era”, was held as part of the 19th Seminário Nacional de História da Ciência e da Tecnologia, from July 29 to August 2, at the Universidade Federal da Bahia, in Salvador. In the second mini-course, “History for whom? Uses of the past and scientific dissemination in history”, Cerqueira taught a class on the importance of popularizing history and science in specialized journals. This mini-course took place December 4 to 6 at the Universidade Federal dos Vales do Jequitinhonha e Mucuri, in Diamantina, Minas Gerais.

Our blog registered over 231,000 page views in 2024. We maintained a consistent rhythm of publications on our social media channels with three to four texts per week, most dedicated to sharing new articles, interviews and events of interest in the fields of history of science, health, and heritage studies. In December 2024, we launched a profile on Bluesky (@revistahcsm.bsky.social), a new platform similar to X (previously Twitter) in terms of use, but without that network’s problems related to scientific denial. Bluesky is widely used among European and North American professors and researchers, promoting interaction with scientists in a variety of knowledge areas. With this additional initiative, *História, Ciências, Saúde – Manguinhos* is expanding its planning of how to internationalize its content.

Another important initiative during the second half was the creation of an editorial committee to reformulate the journal’s editorial body, promoting turnover and partial renewal of the assistant and section editors. It is important to note that this turnover (which includes the scientific editor) is a widely adopted practice at international scientific journals. The objective of this strategy is to revitalize editorial dynamics, incorporate new perspectives, promote greater academic diversity, and expand the impact of published articles.

The first alterations included adding new assistant editors, who make significant contributions to the journal’s reputation: Regina Horta Duarte (https://orcid.org/0000-0003-0808-5435), of the Universidade Federal de Minas Gerais; Maria Isabel Porras (https://orcid.org/0000-0003-2277-6179), of the Universidad de Castilla-La Mancha in Spain; Elizabeth O’Brien (http://orcid.org/0000-0002-4441-274X), of the University of California Los Angeles in the United States; Mauricio Nieto (https://orcid.org/0000-0003-2134-8068), of the Universidad de los Andes in Colombia; and Maria Paula Diogo (https://orcid.org/0000-0003-1504-9248), of the Universidade Nova de Lisboa in Portugal.

Changes were also made to the section editors, such as strengthening the “Divulgação Científica” section, led by Marina Ramalho e Silva (https://orcid.org/0000-0002-2162-6673), which now includes valuable contribution by Carla da Silva Almeida (https://orcid.org/0000-0003-3139-0331), researchers at the Casa de Oswaldo Cruz. We have also combined the responsibility of the “Imagens” and “Fontes” sections, which has now been passed to Professor Mônica Tenaglia (https://orcid.org/0000-0002-5537-0143), of the Universidade Federal Fluminense. We wish to thank all those who have left their roles as members of the Editorial Board but will continue to collaborate with the journal as reviewers or authors: Rafael Huertas, Carlos Henrique Assunção Paiva, Luiz Antônio de Castro Santos, Stefan Pohl-Valero, Charles Monteiro, Luciana Quillet Heymann and Rogério Rosa Rodrigues. To ensure continuity of our high editorial standards, we have opted to maintain some editors in their previous roles. The new assistant and section editors began work on February 15, 2025. An updated list of the editorial staff will be available shortly on the *História, Ciências, Saúde – Manguinhos* website, and we invite our readers to visit.

We are keenly aware that the editorial identity of a scientific journal is one of the main indicators of its quality, and extends beyond its history or the history of the institutions it is associated with. This identity is in fact molded by the academic prestige and dedication of its editorial staff, which are crucial for ensuring the transparency, integrity, and relevance of the published articles. *História, Ciências, Saúde – Manguinhos*’s credibility is reinforced by the fact that Scimago has classified it in the Q2 quartile for Journal & Country Rank in the “History and Philosophy of Science” category, the best ranking in Latin America and surpassing many European publications.

For 2025, we have expansive prospects. During the first weeks of the year, we received an uncommonly large number of submissions, as well as proposals for supplements, which are already being evaluated by reviewers. We are completing a supplement entitled “Colonial collections in Portugal: reconstituting trajectories and rethinking narratives”, organized by Elisabete Pereira, Catarina Simões and Quintino Lopes, which is slated for publication in the second half of 2025. We recently approved two articles that are very relevant for the global history of science, coauthored by internationally acclaimed authors including Kapil Raj, which will certainly help expand the academic impact of our journal.

The *História, Ciências, Saúde – Manguinhos* team is very grateful to all of its authors, reviewers, and editors for their work over the past year. We also acknowledge the support the journal has received from the directorship of the Casa de Oswaldo Cruz, especially its director, Marcos José de Araújo Pinheiro.
