# Supplementary material for: HISTÓRIA, CIÊNCIAS, SAÚDE – MANGUINHOS: CELEBRATING 30 YEARS IN 2024
Source: Hist Cienc Saude Manguinhos. 2025 Jun 16;32:e2025026. doi: 10.1590/S0104-59702025000100026en (PMC12175911; doi:10.1590/S0104-59702025000100026en)
Supplement: Translation

# Translation: HISTÓRIA, CIÊNCIAS, SAÚDE – MANGUINHOS EM 2024: CELEBRAÇÃO DE 30 ANOS

**DOI:** 10.1590/S0104-59702025000100026

**Authors:** Marcos Cueto

**Affiliations:** i Editor científico, pesquisador, Casa de Oswaldo Cruz/Fiocruz. Rio de Janeiro – RJ – Brasil

Como em anos anteriores, apresentamos um resumo das principais atividades da revista em 2024, correspondente ao volume 31 (Cerqueira, Cueto, 2024; Cueto, 2023). Esse período foi marcado pela celebração dos 30 anos da revista, pela continuidade do processo de internacionalização e pelo trabalho de reestruturação do corpo editorial.

Em agosto de 2024, celebramos 30 anos de publicação com um encontro especial intitulado “Ciência aberta e os periódicos científicos de ciências humanas”, realizado nos dias 7 e 8 de agosto, no *campus* da Fiocruz no Rio de Janeiro. O evento teve como foco a discussão sobre ciência aberta, a avaliação de periódicos e a divulgação científica, reunindo editores de revistas do Brasil, México e Chile. As palestras e os debates podem ser assistidos em https://www.youtube.com/watch?v=qYB8V5fbd4U. Um problema identificado na reunião, e um desafio para a área, é a adoção da ciência aberta pelas humanidades. Muitos pesquisadores de ciências humanas encontram dificuldades para adotar as diretrizes da ciência aberta em seus trabalhos.

Continuamos com a publicação em fluxo contínuo pelo segundo ano. Nesse modelo, os textos ficam reunidos em uma única edição, em vez de separados em vários números, como fizemos no passado. Em 2024, *História, Ciências, Saúde – Manguinhos* publicou 74 textos (em todas as seções), sendo 18 deles bilíngues. Em relação à filiação institucional dos primeiros autores dos artigos, continua o predomínio da autoria brasileira, mas tivemos contribuições de Portugal, Espanha, Argentina, Chile, Estados Unidos, México, Uruguai, Peru, Colômbia e Rússia. Entre os artigos, destacamos os textos vinculados ao centenário do Instituto Nacional de Saúde da Mulher, da Criança e do Adolescente Fernandes Figueira, um centro de ensino, atenção e pesquisa vinculado à Fiocruz desde 1970 (Teixeira, Velasquez de Souza, 2024). No segundo semestre de 2024, publicamos o suplemento “A evolução do hospital e a gestão da saúde: entre o local e o global” (https://www.scielo.br/j/hcsm/i/2024.v31suppl1), que apresenta análises aprofundadas e multifacetadas sobre a evolução da gestão hospitalar, tendo sido organizado por editoras convidadas de renome: Isabel Amaral, Alexandra Esteves e Céline Paillette.

Membros de nossa equipe participaram de uma oficina promovida pelo Instituto de Comunicação e Informação Científica e Tecnológica em Saúde, na Fiocruz, em junho de 2024, com o objetivo de aprofundar o entendimento sobre o funcionamento da plataforma Open Journal Systems na publicação científica, cuja implementação realizaremos este ano. Além disso, estivemos presentes em reuniões da Scientific Electronic Library Online (SciELO), da Associação Brasileira de Editores Científicos, e nos Fóruns de Editores da Fiocruz e da Associação Nacional de História. Entre as iniciativas mais relevantes discutidas nessas reuniões, destaca-se o projeto SciELO Acessibilidade (https://www.scielo.br/about/gt-acessibilidade), que propõe mudanças na publicação científica, com o objetivo de torná-la mais inclusiva.

Em colaboração com a Assessoria de Comunicação da Casa de Oswaldo Cruz e a Biblioteca de História das Ciências e da Saúde, contamos com um bolsista dedicado à análise das métricas da revista e à criação de uma metodologia para o monitoramento dos dados do *blog*, bem como das estratégias de divulgação nas redes sociais.

Trabalho de fôlego realizado pela editoria de texto da revista resultou no treinamento de novas revisoras e na atualização dos materiais de referência utilizados no processo de revisão, em paralelo à publicação de artigos. Além disso, implementamos uma nova etapa na preparação final dos originais, que consiste no envio do texto diagramado aos autores para uma última conferência, na busca por reforçar a transparência de nosso processo editorial.

Em julho de 2024, *História, Ciências, Saúde – Manguinhos* submeteu uma proposta de solicitação de auxílio ao Conselho Nacional de Desenvolvimento Científico e Tecnológico e à Coordenação de Aperfeiçoamento de Pessoal de Nível Superior, que foi aprovada para financiamento. Ao longo do ano, Roberta Cerqueira, editora executiva, participou de dois minicursos de relevância. O primeiro, “Historiografia das ciências: produção, metodologias e fontes na era digital”, foi realizado durante 19º Seminário Nacional de História da Ciência e da Tecnologia, ocorrido entre 29 de julho e 2 de agosto 2024, na Universidade Federal da Bahia, na cidade de Salvador. No segundo minicurso, “História para quem? Os usos do passado e divulgação científica em história”, Cerqueira ministrou aula sobre a importância da popularização da história e da ciência em revistas especializadas. Este minicurso aconteceu entre 4 e 6 de dezembro, na Universidade Federal dos Vales do Jequitinhonha e Mucuri, em Diamantina, Minas Gerais.

Nosso *blog* registrou mais de 231 mil visualizações de páginas em 2024. Nossas redes sociais mantiveram um ritmo consistente de publicações, com três a quatro textos por semana, sendo a maioria dedicada à divulgação de novos artigos, entrevistas e eventos de interesse nos campos da história da ciência, da saúde e dos estudos de patrimônio. Em dezembro de 2024, lançamos um perfil na rede social Bluesky (@revistahcsm.bsky.social), uma nova plataforma que se assemelha ao X (antigo Twitter) em termos de uso, mas que não apresenta os problemas de negacionismo científico desta rede. Atualmente, o Bluesky é amplamente utilizado por professores e pesquisadores europeus e norte-americanos, promovendo a interação com cientistas de diversas áreas do conhecimento. Com mais essa iniciativa, *História, Ciências, Saúde – Manguinhos* aprofunda seu planejamento na internacionalização de seus conteúdos.

Uma iniciativa importante no segundo semestre do ano foi a criação de uma comissão de editores, com o propósito de reformular o corpo editorial da revista, promovendo a rotatividade e a renovação parcial dos editores adjuntos e de seção. É importante ressaltar que essa rotatividade, incluindo a do editor científico, é uma prática amplamente adotada por periódicos científicos internacionais. Essa estratégia tem como objetivo revitalizar a dinâmica editorial, integrar novas perspectivas, promover mais diversidade acadêmica e ampliar o impacto dos artigos publicados.

As primeiras alterações incluíram a incorporação de novos editores adjuntos, que contribuem significativamente para o prestígio da revista: Regina Horta Duarte (https://orcid.org/0000-0003-0808-5435), da Universidade Federal de Minas Gerais; Maria Isabel Porras (https://orcid.org/0000-0003-2277-6179), da Universidade de Castilla-La Mancha, Espanha; Elizabeth O’Brien (http://orcid.org/0000-0002-4441-274X), da Universidade da Califórnia em Los Angeles, EUA; Mauricio Nieto (https://orcid.org/0000-0003-2134-8068), da Universidade dos Andes, Colômbia; e Maria Paula Diogo (https://orcid.org/0000-0003-1504-9248), da Universidade Nova de Lisboa, Portugal.

No âmbito dos editores de seções, também foram implementadas mudanças, como o fortalecimento da seção de “Divulgação Científica”, liderada por Marina Ramalho e Silva (https://orcid.org/0000-0002-2162-6673), que agora conta também com a valiosa contribuição de Carla da Silva Almeida (https://orcid.org/0000-0003-3139-0331), pesquisadoras da Casa de Oswaldo Cruz. Além disso, a responsabilidade das seções “Imagens” e “Fontes” foi assumida pela professora Mônica Tenaglia (https://orcid.org/0000-0002-5537-0143), da Universidade Federal Fluminense. Agradecemos aos que agora deixam seus cargos como membros do Corpo Editorial e seguirão colaborando com a revista como avaliadores ou autores: Rafael Huertas, Carlos Henrique Assunção Paiva, Luiz Antônio de Castro Santos, Stefan Pohl-Valero, Charles Monteiro, Luciana Quillet Heymann e Rogério Rosa Rodrigues. Para assegurar a continuidade dos altos padrões editoriais, decidimos manter alguns editores em suas funções. Os novos editores adjuntos e de seção começaram a trabalhar em 15 de fevereiro de 2025. Uma lista atualizada do corpo editorial em breve estará disponível no *site* de *História, Ciências, Saúde – Manguinhos*, e convidamos nossos leitores e leitoras a consultá-la.

Somos cientes de que a identidade editorial de uma revista científica constitui um dos principais indicadores de sua qualidade e vai além de sua história ou das instituições a ela associadas. Ela é, na verdade, moldada pelo prestígio acadêmico e pela dedicação do seu corpo editorial, cruciais para assegurar a transparência, a integridade e a relevância dos artigos publicados. A credibilidade de *História, Ciências, Saúde – Manguinhos* é confirmada pelo fato de o Scimago Journal & Country Rank classificá-la no quartil 2 da categoria “History and Philosophy of Science”, a melhor colocação na América Latina e acima de diversas publicações europeias.

Para 2025, temos grandes perspectivas. Nas primeiras semanas do ano, registramos um volume expressivo e incomum de submissões, além de propostas para suplementos, que já estão em análise pelos pareceristas. Estamos finalizando o suplemento “Coleções coloniais em Portugal: reconstituir trajetórias e repensar narrativas”, organizado por Elisabete Pereira, Catarina Simões e Quintino Lopes, com previsão de publicação no segundo semestre de 2025. Recentemente, aprovamos dois artigos de grande relevância sobre a história global da ciência, escritos em coautoria por pesquisadores de renome internacional, como Kapil Raj, que certamente contribuirão para ampliar o impacto acadêmico da revista.

A equipe de *História, Ciências, Saúde – Manguinhos* fica muito grata aos autores, avaliadores e editores pelo trabalho realizado no ano passado. Reconhecemos o apoio da direção da Casa de Oswaldo Cruz, em especial de seu diretor, Marcos José de Araújo Pinheiro, à revista.
